# Diseases of the Mucous Membrane

**Published:** 1889-10

**Authors:** Griffith P. Terry


### Diseases of Mucous Membranes.

BY GRIFFITH P. TERRY.

This class of membranes which line organs and passages com municating with the exterior of the body, though presenting modifications as to their minute structure in different parts ot the body, exhibit a general resemblance in their construction and consist essentially of submucous tissue; a basement membrane; epithelium of various kinds covering the free surface and numerous glands or follicles differing in their characters in different tracts. They are highly vascular and many of them contain many absorbent vessels. It is only intended in this paper to take up briefly, from a general point of view, the morbid conditions to which mucous structures as a class are exposed. They are:

1. Injury.—Most of the mucous membranes are exposed to injury from various causes. This may come from without, the cause being either mechanical, chemical, or excessive heat, such as injury to the mucous lining of the alimentary canal or air passages by foreign bodies; corrosion from swallowing strong acids, and burning or scalding the mouth, or of parts lower down in consequence of inhaling a hot blast or swallowing boiling water. In other cases the injury may arise from within the body—such as parasites, the rupture of enlarged veins, aneurisms, or abscesses opening into mucous cavities. The effects of an injury to a mucous surface differ in their nature and extent according to its cause. Thus there may be a mere contusion, a superficial erosion or abrasion, a more or less extensive wound or rupture, other structures being then also involved, a burn or scald, or actual destruction by corrosives. More or less inflammation follows injury to a mucous membrane. Subsequently ulcers may be produced, which by their cicatrization give rise to constriction or actual obliteration of tubes and other structures.

2. Hyperæmia and Anæmia.—The mucous membranes are very prone to become the seat of congestion either active, mechanical, or passive. Active congestion may be a part of a physiological action as in the gastric mucous membrane during digestion. Any slight irritation may also cause it, and it is hardly possible to distinguish this condition from that of inflammation of which active congestion forms the earliest stage. It is characterized by bright redness, new vessels frequently coming to view; and at first by a tendency to dryness of the affected membrane which may be followed by excessive and altered secretion. Mechanical congestion is often an important morbid condition in connection with these structures, giving rise to troublesome symptoms. For instance, in cases of cardiac disease obstructing the pulmonary circulation, the mucous lining of the airpassages becomes more or less congested permanently; and if the general venous circulation becomes overloaded from a similar cause, other mucous tracts suffer, especially that of the alimentary canal. Particular portions of a mucous membrane might become the seat of mechanical congestion, if some local vein should become obstructed from any cause. The effects of this condition are first to make the color deeper, with a more or less venous hue, and at last the small veins may be evidently dilated and varicose. The secretions become modified in quality and quantity, and in time a permanent discharge is likely to be established, consisting of an unhealthy, thick, and tenacious mucous while the proper secretion of special glands is interfered with, such as the gastric juice. In some instances mechanical congestion gives rise to an abundant flow of a watery mucus. The membrane itself is also liable to become altered, being swollen at first, and ultimately it may become permanently thickened and firmer than normal, owing to increase of connective tissue, while its own special structures degenerate.

Passive hyperæmia may follow inflammation of a mucous membrane; or it occurs in persons of relaxed and feeble habit or follows undue use of a part covered with a mucous membrane, as in the case of the throat.

Anæmia in connection with a mucous membrane is important only when this is a part of general anæmia from any cause. Those mucous surfaces which are visible, such as the conjunctiva or the lining of the mouth and lips, give the most striking evidence of this condition, as indicated by their pallor or even bloodlessness. An anæmic condition of the alimentary canal interferes quite considerably with the function of its mucous membrane and with the formation of the secretions which it normally produces.

3. Inflammation.—Various forms and degrees of inflammation are commonly met with in mucous membranes pertaining to the acute, subacute, or chronic forms, and either catarrhal, croupous, or diphtheric in character. Different tracts of membrane present different degrees of liability to these several forms of inflammation, and the catarrhal form not only has various grades of intensity, with corresponding variety in its products, which may become muco-purulent or actually purulent, but these products also differ in their nature in connection with different membranes of the mucous class. Further inflammation from special causes is characterized by running a special or definite course and forming special products. When the inflammation is of a severe type it may end in more or less destruction of the mucous tissues, as indicated by erosions, ulceration, or even gangrene. Where the submucous tissue is loose œdema is liable to occur. From this cause as well as from thickening of the mucous membrane itself, or from a croupous or other deposit on its surface, narrowing or even actual closure of any tube or passage lined by such a membrane is apt to be produced. Inflammation may also give rise to submucous suppuration. When the inflammation is chronic permanent changes are brought about in mucous tissues, the normal elements being altered or entirely removed and a fibroid material being formed in course of time, so that the membrane is rendered permanently thickened and tough. The cause of inflammation of a mucous membrane may be local, including injury, mechanical or chemical irritation (stimulation), or that resulting from excessive heat or cold, morbid products or growths, or *general*, such as chilling of the body from "a cold," blood poisoning in connection with fevers and other conditions; or the inflammation may be a part of some specific disease, such as diphtheria. Some mucous tracts are particularly liable to be affected under certain predisposing conditions and at certain periods of life. Thus bronchitis is very common in children and old persons, while the former are exceedingly subject to catarrh of the alimentary mucous lining.

4. ULCERATION.—Ulcers are of common occurrence on mucous surfaces. They usually result from injury or inflammation, or are the termination of certain morbid processes as in the case of typhoid fever, syphilis, cancer. Ulceration may depend upon destruction of the tissues by parasitic growths as in "thrush." Some pathologists are of the opinion that ulceration of a mucous membrane occasionally arises from plugging of arteries and consequent death of a limited portion of this membrane, which separates leaving an ulcer. In the case of the stomach it has also been supposed that under certain circumstances the gastric juice may so act upon the mucous lining as to destroy it. A peculiar form of ulcer is sometimes observed after severe burns. Ulceration often begins in connection with the glandular structures; this may be due to mere blocking up of their orifices leading to accumulation of their products and subsequent inflammation, but it is believed that morbid processes commence in these tissues. Inflammation may cause ulceration either by directly destroying the membrane or gradually; or by setting up submucous suppuration. Mucous ulcers differ much in their seat extent, depth, shape, and other characters according to their cause. The simple forms are either mere erosions, or of the catarrhal or follicular varieties, and in each of the special diseases (diseases already mentioned) the ulcers present peculiar characters. Occasionally they assume a gangrenous appearance. If they extend deeply they involve other tissues besides those of the mucous membrane and may thus lead to perforation of cavities, or tubes, etc. Cicatrization often takes place and this may lead to permanent contraction, stricture, or even complete closure of channels lined by mucous membrane with more or less thickening and induration. Ulceration frequently destroys glandular structures which are not renewed afterward.

5. Gangrene.—Occasionally the tissues forming mucous membrane mortify as the result of either severe injury, corrosion, inflammation, and vascular obstruction. The gangrene is of the moist kind and the dead tissue may separate in a mass or in shreds. Consequently an ulcer is left, or actual perforation of a tube or hollow organ may take place.

6. Nutritive Changes.—Hypertrophy of mucous tissue is sometimes seen, but this may appear to be the case when it is really not so, the membrane being thickened and firm owing to a chronic inflammation, the formation of fibrous tissue. Atrophy is not uncommon, especially of certain of the elements of mucous membranes, such as the glands or epithelium. Degeneration is also often observed affecting these structures. This degeneration may be of a senile character, or of a special kind, such as albuminoid or mucous degeneration. Not uncommonly mucous tissues are relaxed and deficient in tone, their nutrition being impaired.

7. Deposits and New Growths.—The main new formations observed in connection with mucous membranes are polypi, villous growths, epithelioma, and tubercle. Siphilitic gummata may involve these membranes. Cysts also occasionally form, originating from the glands or epithelial structures; it is claimed also that certain animal and vegetable parasites are often associated with mucous membranes.

8. Special Diseases.—In certain diseases mucous membranes are particularly affected, such as typhoid fever, diphtheria, and dysentery.

Treatment.—The general principles or indications in the treatment of diseases of the mucous membranes may be summed up as follows: Relieve pain and other sensations with appropriate means; check hemorrhages if they are in such amount as to need interference; subdue inflammatory action; brace up their tone to the tissues relaxed; influence secretions and mordid products, increasing or diminishing the former, checking or modifying discharges and endeavoring to affect certain materials, such as diphtheretic deposits; allay undue excitability causing violent actions, or aid such actions as may be necessary to expell excessive secretions or morbid products, or rather, to prevent their accumulation; supply the place of and prevent the symptoms resulting from the want of secretions necessary for special purposes which are formed by certain mucous surfaces, such as the gastric juice; treat particular morbid conditions, such as ulcers, gangrene, new growths, or constriction, with the view of curing them.

Treat General Symptoms.—Local applications, or such remedies as when administered internally come into contact with the affected surface, are of much value, which may be anodyne, sedative, stimulating, astringent, demulcent, or of other kinds according to the action required; and they are often applied advantageously in special ways. Operations are not infrequently required; general treatment is often of most service in the management of these diseases, and may be the only indications, yet there are certain diseases in which the morbid conditions of the mucous membranes is but a part of the general malady and calls for no special treatment.

---

Aphorisms for Emergencies.—When dust gets into the eyes avoid rubbing with the fingers, but dash cold water into them. Remove cinders with a camel's hair pencil. Remove water from the ear with warm water. Never use a probe or other hard substance for the ear lest you perforate the drum.

